# Data subdivision approach enhances machine learning-based mortality prediction in pediatric ICU patients

**DOI:** 10.1371/journal.pone.0349772

**Published:** 2026-06-16

**Authors:** Wenqian Chen, Benjamin Lee, Zexi Zang, Junfeng Li, Lingna Huang, Hang Xing, Yanli Ren

**Affiliations:** 1 Department of Neonatology, Fujian Maternity and Child Health Hospital, College of Clinical Medicine for Obstetrics & Gynecology and Pediatrics, Fujian Medical University, Fuzhou, Fujian, China; 2 Department of Pediatrics, Women & Infants Hospital of Rhode Island, Warren Alpert Medical School of Brown University, Providence, Rhode Island, United States of America; 3 Henan Key Laboratory of Fertility Protection and Aristogenesis, Department of Reproductive Center, Luohe Central Hospital, Luohe, Henan, China; 4 Department of Gynecology and Obstetrics, Fujian Maternity and Child Health Hospital, College of Clinical Medicine for Obstetrics & Gynecology and Pediatrics, Fujian Medical University, Fuzhou, Fujian, China; Najran University College of Computer Science and Information Systems, SAUDI ARABIA

## Abstract

**Objective:**

To evaluate machine learning–based models for predicting all-cause mortality in pediatric ICU patients using comprehensive biochemical panels, with a focus on addressing missing data and class imbalance.

**Materials and methods:**

A retrospective analysis was performed on a publicly available PICU dataset comprising 8,629 patients aged 28 days to 18 years. Twenty-two biochemical variables measured on the first PICU day were analyzed. Missing values were addressed using Multiple Imputation. Lasso regression was applied for feature selection. Models were trained using 5-fold cross-validation with the Synthetic Minority Oversampling Technique (SMOTE). Two ML-based imbalance-handling strategies, stacking ensemble and data subdivision were evaluated. Pairwise DeLong tests were used to compare AUC performance across models.

**Results:**

Among the 8,629 included patients, there were 476 non-survivors (5.5 percent). Multiple imputation followed by SMOTE improved model performance across all algorithms. The single-model classifiers achieved AUC-ROC values of 0.82 (Random Forest), 0.79 (CatBoost), 0.83 (Extra Trees), and 0.79 (Logistic Regression). The stacking ensemble demonstrated the best overall performance, with an AUC-ROC of 0.88 and an AUC-PRC of 0.45. The data subdivision approaches also produced strong discriminative performance, achieving AUC-ROC value up to 0.83 for three-subdivision, and 0.82 for five-subdivision strategies. Calibration analysis showed that the stacking model achieved the lowest Brier score (0.04), indicating superior probabilistic accuracy compared with individual classifiers. Feature importance analyses across all MI-based models consistently highlighted coagulation markers (D-dimer, reference TT, PTT, INR), electrolytes (chloride, potassium, sodium), and metabolic and organ-dysfunction indicators (AST, ALT, creatinine) as key predictors of mortality.

**Conclusions:**

This study demonstrates that ensemble stacking is a more effective strategy than data subdivision for addressing class imbalance in PICU mortality prediction.

## Introduction

Children admitted to pediatric intensive care unit (PICU) with critical illnesses are at high risk of clinical deterioration and subsequently increased risk of mortality [1]. Therefore, PICUs are designed to provide specialized critical care and continuous monitoring for severely ill children. Significant evidence has suggested that biochemical and clinical warning signs can help predict the risk of mortality in PICU [2,3]. Currently, the prevalence of all-cause mortality in PICU ranges from 6.7% to 49.8% [4–6]. Over the years, prognostic scoring systems such as the Pediatric Risk of Mortality (PRISM) and the Pediatric Index of Mortality (PIM) have been widely adopted in PICUs, with their latest versions being PRISM 4 and PIM 3 [7]. In addition, the Pediatric Logistic Organ Dysfunction 2 (PELOD 2) score, designed for daily assessment of organ dysfuction, has been highly predictive of mortality in children with suspected infection. [8,9]. More recently, the Phoenix Sepsis Criteria have demonstrated improved performance in diagnosing pediatric sepsis and septic shock compared to the previous International Pediatric Sepsis Consensus Conference criteria for sepsis [10]. According to other studies, these scoring systems have demonstrated the receiver operating characteristic (ROC) area under the curve (AUC) values ranging from 0.667 to 0.96, indicating moderate to strong predictive performance [2,10–16]. However, their predictive performance appears to be lower in regions outside North America and Europe, potentially due to differences in healthcare infrastructure, patient demographics, and resource availability [17].

The implementation of machine learning (ML) in healthcare has opened several avenues of research and its clinical applications. ML can transform predictive modeling by analyzing vast amounts of data and providing risk prediction for mortality in PICUs [7,8]. Furthermore, ML algorithms have superior efficacy compared to traditional scoring systems as these algorithms can process dozens of variables simultaneously [9]. In the PICU, routine blood tests are essential for monitoring disease progression and guiding treatment. However, patients in ICU frequently receive extensive panels of routine diagnostic tests, many of which are conducted solely for monitoring potential complications without a clear clinical indication [10]. One study suggests that up to 67.9% of laboratory test results do not significantly contribute to patient management, raising concerns about unnecessary costs and excessive blood loss from repeated phlebotomy [11]. Extracting predictive models from clinical data is quite challenging due to irregularity of measurements and temporal nature of data, but ML can be effective in such cases as it can handle clinical data complexity compared to traditional models [12].

Previous studies have shown the efficacy of ML models in predicting mortality risk in pediatric patients. A study by Hu et al. involving 1481 neonates, investigated the efficacy of ML models in predicting risk factors for postoperative mortality [13]. Their results showed that random forest model achieved the best performance with an AUC value of 0.72. The models not only predicted mortality but also explained risk factors in each case [13]. Similarly, another study reported an AUROC score of 0.71 in predicting the risk of readmission of neonates in ICU [14]. While many studies have focused on predicting mortality for specific diseases or conditions, limited research has addressed all-cause mortality prediction in PICUs. This gap highlights the need for comprehensive models capable of capturing the diverse causes of mortality encountered in PICU.

However, training ML-based predictive models in clinical settings presents several challenges, including limited availability of publicly accessible datasets, high data variability, and severe class imbalance. Many ICU datasets are highly imbalanced, where non-survivors are significantly underrepresented compared to survivors. In such datasets, models tend to favor the majority class, reducing sensitivity and limiting their ability to accurately identify high-risk patients. To address these challenges, this study investigates advanced data processing techniques, including multiple imputation, synthetic minority oversampling (SMOTE), stacking ensemble methods, and data subdivision approaches to mitigate data missing and class imbalance and evaluate its impact on model development and performance.

## Materials and methods

### Data collection and processing

This study utilized a publicly available PICU dataset, consisting of 12,881 patient records collected between 2010 and 2019 at The Children’s Hospital of Zhejiang University School of Medicine. To ensure patient privacy, all identifiers required by the Health Insurance Portability and Accountability Act (HIPAA) were removed, resulting in fully de-identified data. This study is based on publicly available database and do not require additional ethical approval or informed consent.

**Fig 1:**
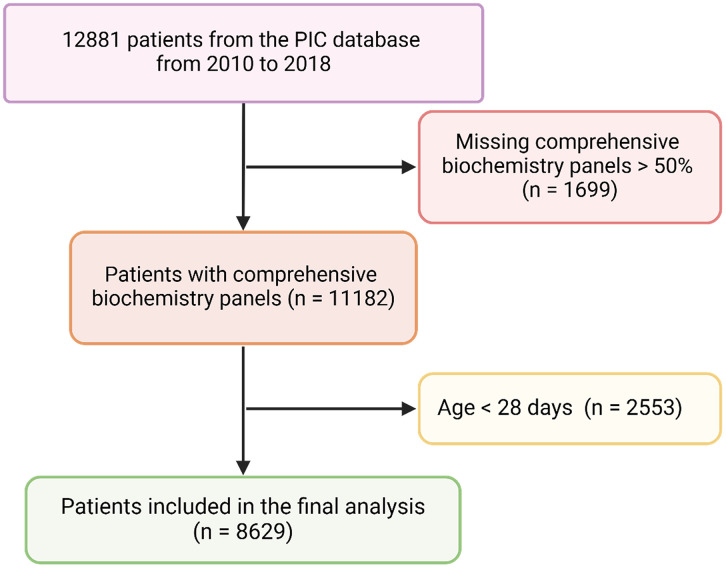
Study population selection process. Initial 12,881 PICU patient records, with exclusions for >50% missing data and neonates <28 days, resulting in 8,629 records for analysis.

After applying inclusion and exclusion criteria, a total of 8,629 patient records were retained. Patients were excluded if more than 50% of data were missing (n = 1,699) or if they were neonates aged 28 days or younger (n = 2,553). After applying exclusion criteria, a total of 8,629 patient records were included in the study, consisting of 8,153 survivors (94.5%) and 476 non-survivors (5.5%), reflecting a highly imbalanced class distribution. Fig 1 illustrates the detailed study population.

The dataset originally contained 22 variables, including demographic information such as age and gender, as well as blood biochemistry parameters measured on the first day of PICU admission. These parameters were categorized into different physiological panels: lipid and glucose markers (triglycerides, total cholesterol, glucose), electrolytes (potassium, chloride, sodium, calcium), coagulation markers (partial thromboplastin time (PTT), international normalized ratio (INR), reference prothrombin time (PT), reference thrombin time (TT), D-dimer, functional fibrinogen), and liver and kidney function markers (alanine aminotransferase (ALT), aspartate aminotransferase (AST), albumin, total bilirubin, total protein, creatinine, globulin).

### Model training

**Fig 2:**
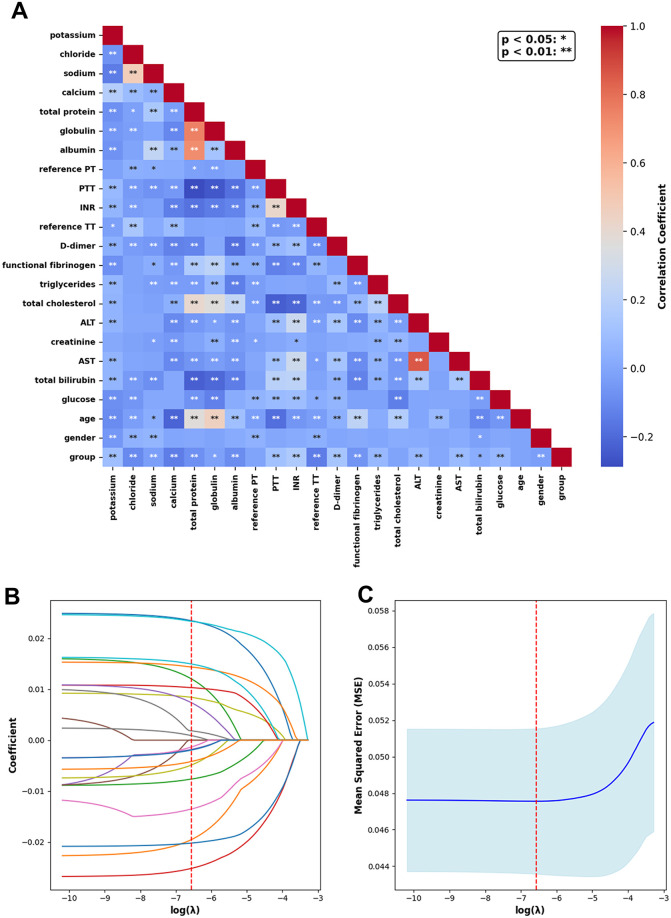
Feature selection using Lasso regression. **A** illustrates the pairwise correlation matrix of all features included in this study. **B** shows the coefficient profiles of selected clinical features, where the vertical dashed line represents the chosen λ value on a logarithmic scale. **C** shows the variation of the hyperparameters λ plotted against mean squared error (MSE), with light-red vertical lines indicating optimal values based on the one-standard-deviation criterion.

The refined PICU data was divided into model development (70%) and model internal testing (30%) datasets using stratified random sampling for approximately equal frequencies of survivors and non-survivors in both subsets. To optimize the predictive model, missing data of development dataset were first addressed using MI via chained equations framework, which preserves the multivariable relationships within the dataset and minimizes information loss prior to model development. Five imputed datasets were generated ensuring appropriate estimation for both continuous and categorical variables. Following imputation, Lasso regression was applied to address multicollinearity, reduce overfitting, and select a parsimonious set of features. Feature selection was based on non-zero coefficients identified using the one-standard-error rule during cross-validation, and no additional filtering criteria were applied during this process. Fig 2B–C illustrates the feature selection process based on Lasso regularization, showing model coefficients shrink as the penalty parameter increases and the optimal λ value was determined through cross-validation.

Four classification models were trained on the development subset: Logistic Regression, Random Forest, CatBoost, and Extra Trees. Among them, Logistic Regression was implemented as the conventional clinical baseline model to facilitate comparison with other complex machine learning approaches. To enhance model reliability and mitigate overfitting, each model underwent a 5-fold cross-validation procedure. Synthetic Minority Oversampling Technique (SMOTE) was applied within each fold to oversample the minority class to achieve a balanced distribution during training. Hyperparameter tuning was then performed using a random grid search strategy to select the optimal hyperparameters (S1 Table). This cross-validation process was repeated five times to ensure each fold served as the validation set exactly once, enhancing the reliability of performance estimates. For each validation fold, AUROC was calculated to assess the model’s discriminatory ability.

Upon completion of cross-validation, the model with the highest validation AUC was selected, and its corresponding hyperparameters were saved. The holdout testing dataset was processed using the Multiple Imputation transformer to handle missing values. Subsequently, the saved model was evaluated on this test set, where AUC-ROC was again computed, and the optimal prediction threshold determined during validation was applied to predict outcomes. To comprehensively assess the model’s performance, key evaluation metrics including AUC-ROC, AUC-PRC, Precision, Sensitivity, Specificity, Accuracy, and F1-Score were calculated. The area under the precision-recall curve (AUC-PRC) was reported to better reflect performance under class imbalance. Finally, the model’s generalizability was assessed using an internal validation dataset.

### Addressing class imbalance

Given the highly imbalanced nature of the dataset, where the majority class (survivors, n = 8153) significantly outnumbers the minority class (non-survivors, n = 476), two strategies were employed to mitigate class imbalance and improve predictive performance: a stacking ensemble method and a data subdivision approach. Both strategies aimed to enhance the model’s ability to learn from a poorly balanced distribution of outcomes to improve its predictive performance.

### Stacking ensemble method

Ensemble Learning method refers to a learning methodology that combines multiple single base models to create a single model that is more powerful than any individual model alone [25,26]. A stacking ensemble method, which is one of the popular ensemble learning methods, has been widely used in machine learning to mitigate class imbalance problems [27].

**Fig 3:**
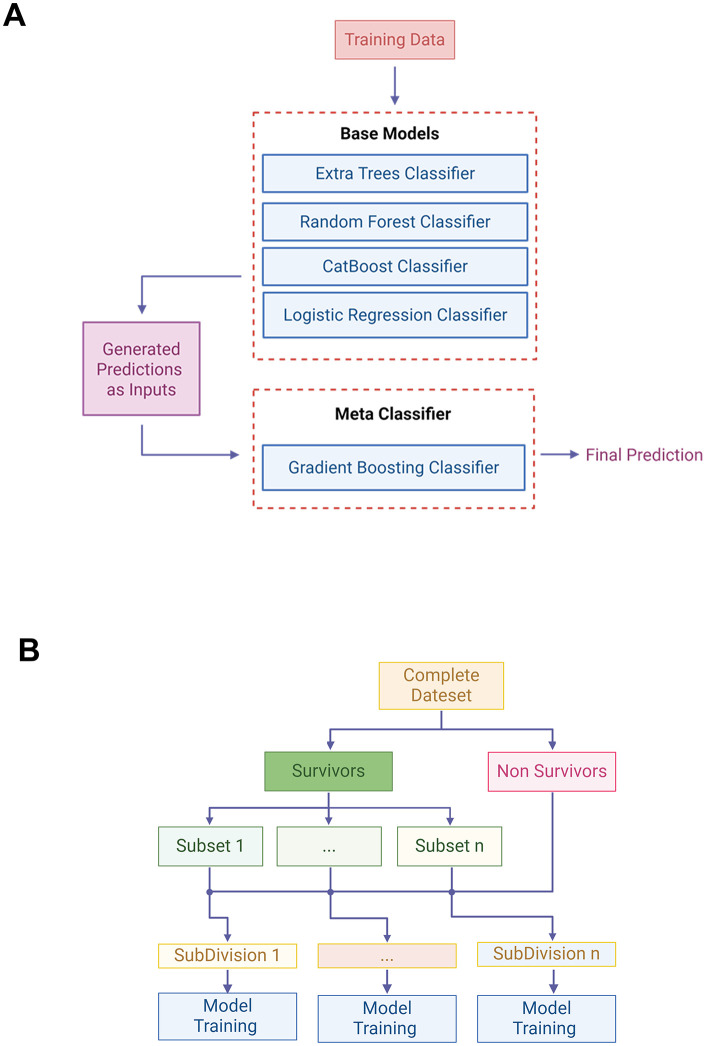
Model architectures for handling class imbalance. **A** Stacking Ensemble technique flowchart that illustrates how predictions from six base models are combined using a gradient-boosting meta-classifier to generate the final prediction. **B** n-subdivision method flowchart that illustrates how the majority class is divided into subsets and aggregated to enhance class balance and model generalizability.

In this approach, the same six base models were used to generate initial predictions, which were then combined using a Gradient Boosting meta-classifier. Fig 3A illustrates the stacking ensemble framework. To maintain class balance during training, 5-fold cross-validation and SMOTE were applied as well to ensure the minority class was adequately represented in each fold. Subsequently, hyperparameter tuning was conducted as described earlier, to identify the optimal model parameters. Upon completion of cross-validation, the model with the highest validation AUC was selected, and its corresponding hyperparameters were saved.

### Data subdivision method

The data subdivision method was explored as an alternative approach to mitigating class imbalance. Instead of training models on the complete dataset, the majority class (survivors) was divided into n-number of subsets, while the minority class (non-survivors) was oversampled using SMOTE to match the number of cases in each majority class subset. Each subset from the majority class was then combined with the same oversampled minority class to form distinct subdivisions. By doing so, each subdivision has an equal number of survivors and non-survivors and thus maintains a consistent and balanced representation of the minority class across all subdivisions. In this paper, three-subdivision and five-subdivision methods were investigated to evaluate which strategy yields better predictive performance. **Fig 3B** illustrates the data subdivision technique used in this study, showing how the dataset was partitioned into smaller subsets and combined to create more balanced training dataset, ultimately improving predictive performance.

For the three-subdivision method, the 70% of majority class (n = 5,707) was divided into three equal subsets of 1,903 cases each. The 70% of minority class (n = 333) was first oversampled using SMOTE to 1,903 cases, matching the size of each majority class subset. Each of the three majority class subsets was then combined with the same oversampled minority class, resulting in three balanced subdivisions. Similarly, for the five-subdivision method, the majority class was divided into five subsets consisting of 1,142 cases each. The minority class was oversampled using SMOTE to 1,142 cases to match the size of each majority class subset, forming five balanced subdivisions.

Each subdivision was independently trained using four base models: Logistic Regression, Random Forest, CatBoost, and Extra Trees. After traning, the model with the highest validation AUC was selected. The predictions generated from all subdivisions were averaged to produce the final prediction.

### Software and statistics

All analyses were performed using Python version 3.12.6. A complete list of software packages and version numbers used in data preprocessing, imputation, model development, and evaluation is provided in the S2 Table. The characteristics of the survivor and non-survivor groups were compared using the χ² test for categorical variables and t-test for continuous variables. Model classification performance was evaluated using AUROC, AUPRC, precision, sensitivity, specificity, accuracy, and F1-score. Pairwise DeLong tests were conducted to statistically compare AUC performance across individual models, the stacking ensemble, and models developed using the three- and five-subdivision approaches. A p-value of less than 0.05 was considered statistically significant for all comparisons.

## Results

### Data description

Of the collected data, the mean age of the overall cohort was 3.2 ± 3.8 years, with no significant difference observed between the survivor (3.2 ± 3.8 years) and non-survivor (3.0 ± 3.9 years) groups (p = 0.419). Male patients comprised 56.0% of the total cohort, with a significantly higher proportion in the non-survivor group (62.0%) compared to the survivor group (55.7%) (p = 0.008). Among the comprehensive biochemistry panel variables, most exhibited statistically significant differences between survivors and non-survivors. Table 1 provides a summary of the descriptive statistics for all features included in this study, presenting key statistical measures such as the class-wise mean, standard deviation, and P-values for all biochemical markers and continuous variables.

**Table 1:** Clinical characteristics of patients.

Features	Missing	Overall (*n* = 8,629)	Survived (*n* = 8,153)	Deceased (*n* = 476)	*P*-Value
Age, mean (SD)	0	3.2 (3.8)	3.2 (3.8)	3.0 (3.9)	0.419
Gender					0.008
Male, *n* (%)	0	4836 (56.0)	4541 (55.7)	295 (62.0)	
Female, *n* (%)	0	3793 (44.0)	3612 (44.3)	181 (38.0)	
Triglycerides, mean (SD)	782	1.2 (1.5)	1.1 (1.4)	1.6 (2.1)	<0.001
Total cholesterol, mean (SD)	782	3.2 (1.2)	3.2 (1.2)	3.1 (1.8)	0.567
Glucose, mean (SD)	388	8.0 (3.9)	8.0 (3.6)	9.1 (7.0)	0.001
Potassium, mean (SD)	4	3.7 (0.7)	3.7 (0.7)	4.0 (1.2)	<0.001
Chloride, mean (SD)	2	108.5 (6.2)	108.7 (6.0)	106.0 (8.0)	<0.001
Sodium, mean (SD)	2	137.7 (5.2)	137.7 (5.1)	137.0 (7.1)	0.034
Calcium, mean (SD)	2	1.2 (0.1)	1.2 (0.1)	1.1 (0.2)	<0.001
PTT, mean (SD)	662	38.4 (19.1)	37.9 (18.3)	47.6 (28.9)	<0.001
INR, mean (SD)	613	1.2 (0.5)	1.2 (0.5)	1.5 (1.1)	<0.001
Reference PT, mean (SD)	591	11.8 (0.4)	11.8 (0.4)	11.8 (0.4)	0.248
Reference TT, mean (SD)	2565	19.0 (0.8)	19.0 (0.8)	18.5 (0.9)	<0.001
D-dimer, mean (SD)	2679	2.5 (3.9)	2.4 (3.7)	5.1 (6.4)	<0.001
Functional fibrinogen, mean (SD)	2589	2.3 (1.1)	2.3 (1.0)	1.9 (1.3)	<0.001
ALT, mean (SD)	184	69.2 (343.5)	63.3 (312.6)	170.5 (671.1)	0.001
AST, mean (SD)	573	148.6 (794.2)	131.8 (712.5)	421.5 (1602.0)	<0.001
Albumin, mean (SD)	576	37.1 (6.1)	37.2 (5.9)	34.8 (8.3)	<0.001
Total bilirubin, mean (SD)	761	20.9 (42.2)	20.6 (41.1)	25.1 (57.1)	0.099
Creatinine, mean (SD)	219	52.8 (288.8)	52.4 (296.9)	58.7 (47.8)	0.116
Globulin, mean (SD)	582	20.4 (6.6)	20.5 (6.6)	19.8 (7.3)	0.047
Total protein, mean (SD)	572	57.4 (9.7)	57.6 (9.5)	54.3 (12.4)	<0.001

### Individual model performance & stacking ensemble

**Fig 4:**
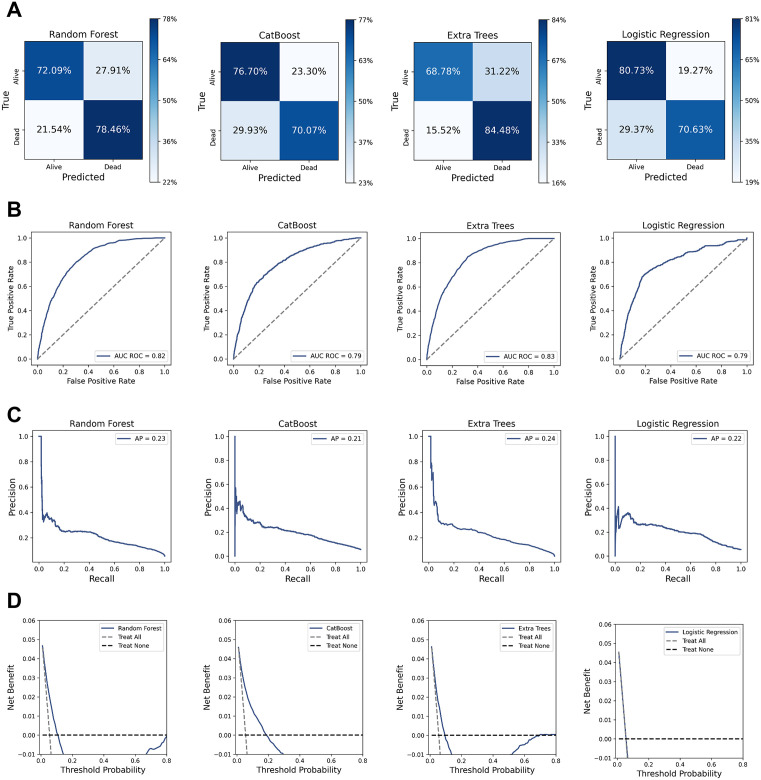
Confusion matrix (A), ROC curve (B), precision/recall curve (C) and decision curve analyses (D) for single models.

**Fig 5:**
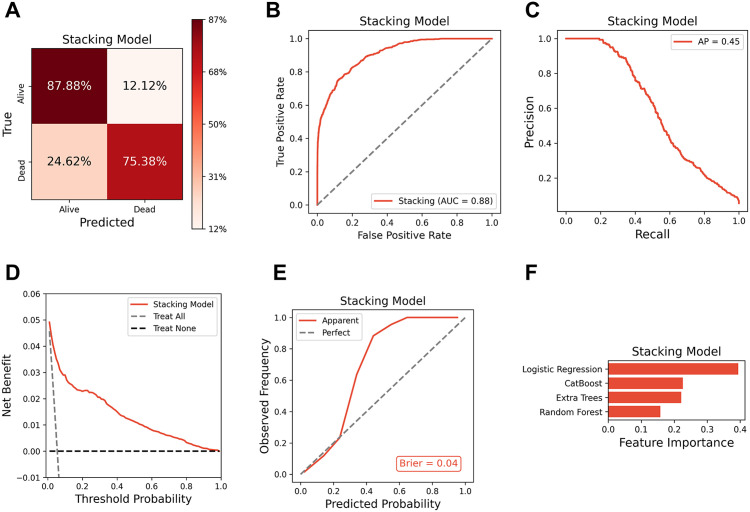
Confusion matrix (A), ROC curve (B), precision/recall curve (C), decision curve analyses (D), calibration curve (E) and feature importance ranking (F) for stacking ensemble model trained with Gradient Boosting.

When trained individually, the single models demonstrated AUROC values of 0.82, 0.79, 0.83, and 0.79 for Random Forest, CatBoost, Extra Trees and Logistic Regression, respectively, with corresponding AUPRC values of 0.23, 0.21, 0.24, and 0.22. These metrics indicate that all four algorithms achieved reasonably strong discriminative performance, with tree-based methods Random Forest, Extra Trees slightly outperforming Logistic Regression. The stacking ensemble, built with Gradient Boosting as the meta-classifier, achieved the highest overall performance among all models. It reached an AUROC of 0.88 and an AUPRC of 0.45, reflecting a substantial improvement in both discrimination and precision–recall performance compared with any individual classifier (Table 2, Figs 4A–C and 5A–C, and S2A). In addition, decision curve analysis showed that the stacking model provided consistently greater net clinical benefit across a wide range of threshold probabilities compared with the treat-all and treat-none strategies, further highlighting its practical utility in clinical decision-making (Figs 4D and 5D).

**Table 2:** Model performance of single model, stacking model, three-subdivision, and five-subdivision approaches.

Type	Model	Accuracy (95 CI)	Precision (95 CI)	Sensitivity (95 CI)	Specificity (95 CI)	F1 Score (95 CI)	AUC-ROC (95 CI)	AUC-PRC (95 CI)
Single model	Random forest	0.72 (0.63, 0.82)	0.14 (0.11, 0.18)	0.79 (0.63, 0.94)	0.72 (0.62, 0.83)	0.24 (0.19, 0.29)	0.82 (0.79, 0.85)	0.23 (0.17, 0.29)
	CatBoost	0.76 (0.66, 0.86)	0.15 (0.10, 0.20)	0.70 (0.59, 0.81)	0.77 (0.66, 0.88)	0.25 (0.18, 0.31)	0.79 (0.76, 0.83)	0.21 (0.15, 0.27)
	Extra trees	0.70 (0.65, 0.74)	0.14 (0.11, 0.16)	0.84 (0.76, 0.93)	0.69 (0.64, 0.74)	0.24 (0.20, 0.27)	0.83 (0.80, 0.86)	0.24 (0.18, 0.30)
	Logistics regression	0.80 (0.76, 0.85)	0.18 (0.14, 0.22)	0.71 (0.62, 0.79)	0.81 (0.76, 0.86)	0.28 (0.23, 0.33)	0.79 (0.75, 0.83)	0.22 (0.16, 0.27)
Stacking model	Gradient boosting	0.86 (0.85, 0.87)	0.24 (0.22, 0.26)	0.71 (0.67, 0.75)	0.87 (0.86, 0.88)	0.36 (0.33, 0.38)	0.88 (0.87, 0.89)	0.45 (0.38, 0.52)
Three-subdivision	Random forest	0.72 (0.64, 0.80)	0.33 (0.25, 0.40)	0.81 (0.81, 0.82)	0.70 (0.61, 0.80)	0.47 (0.39, 0.54)	0.83 (0.78, 0.88)	0.46 (0.41, 0.51)
	CatBoost	0.71 (0.59, 0.83)	0.32 (0.22, 0.42)	0.81 (0.75, 0.86)	0.69 (0.56, 0.82)	0.46 (0.34, 0.57)	0.82 (0.74, 0.90)	0.48 (0.40, 0.56)
	Extra trees	0.68 (0.49, 0.86)	0.31 (0.17, 0.44)	0.85 (0.71, 1.00)	0.64 (0.40, 0.89)	0.45 (0.33, 0.57)	0.83 (0.78, 0.87)	0.48 (0.42, 0.55)
	Logistics regression	0.71 (0.70, 0.73)	0.30 (0.28, 0.32)	0.72 (0.59, 0.85)	0.71 (0.68, 0.74)	0.43 (0.39, 0.47)	0.76 (0.70, 0.83)	0.41 (0.28, 0.55)
Five-subdivision	Random forest	0.74 (0.68, 0.81)	0.46 (0.39, 0.54)	0.76 (0.68, 0.84)	0.74 (0.65, 0.83)	0.57 (0.52, 0.63)	0.82 (0.77, 0.86)	0.56 (0.47, 0.64)
	CatBoost	0.73 (0.64, 0.83)	0.46 (0.34, 0.58)	0.79 (0.72, 0.86)	0.72 (0.57, 0.86)	0.58 (0.50, 0.65)	0.81 (0.77, 0.86)	0.58 (0.49, 0.67)
	Extra trees	0.73 (0.62, 0.83)	0.45 (0.35, 0.55)	0.81 (0.68, 0.94)	0.70 (0.54, 0.87)	0.58 (0.50, 0.65)	0.82 (0.77, 0.88)	0.58 (0.50, 0.65)
	Logistics regression	0.74 (0.69, 0.78)	0.45 (0.39, 0.51)	0.66 (0.49, 0.84)	0.76 (0.65, 0.86)	0.53 (0.49, 0.57)	0.75 (0.70, 0.81)	0.51 (0.44, 0.59)

### Performance of the data subdivision approach

**Fig 6:**
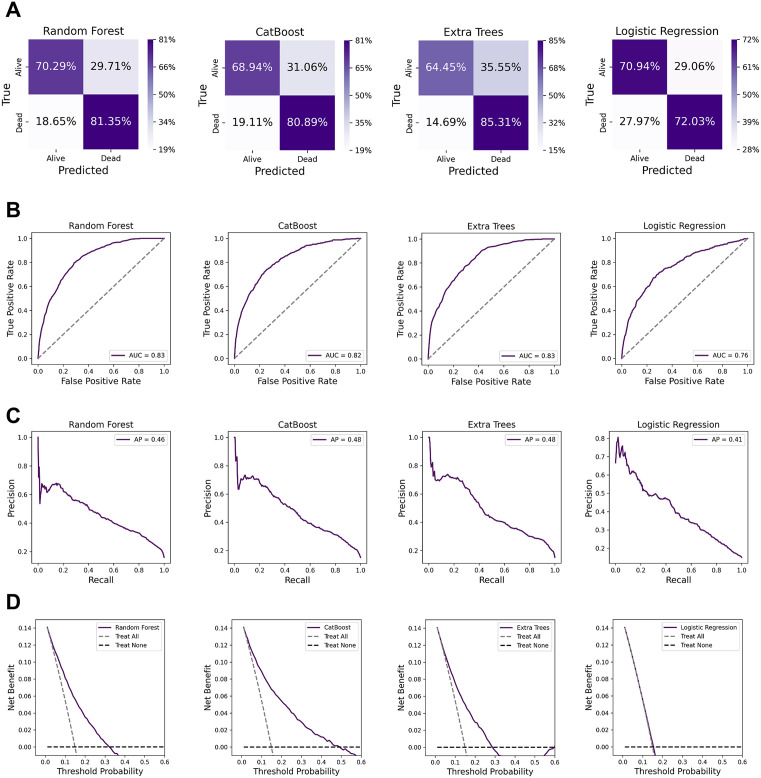
Confusion matrix (A), ROC curve (B), precision/recall curve (C) and decision curve analyses (D) for single models using the three-subdivision approach.

**Fig 7:**
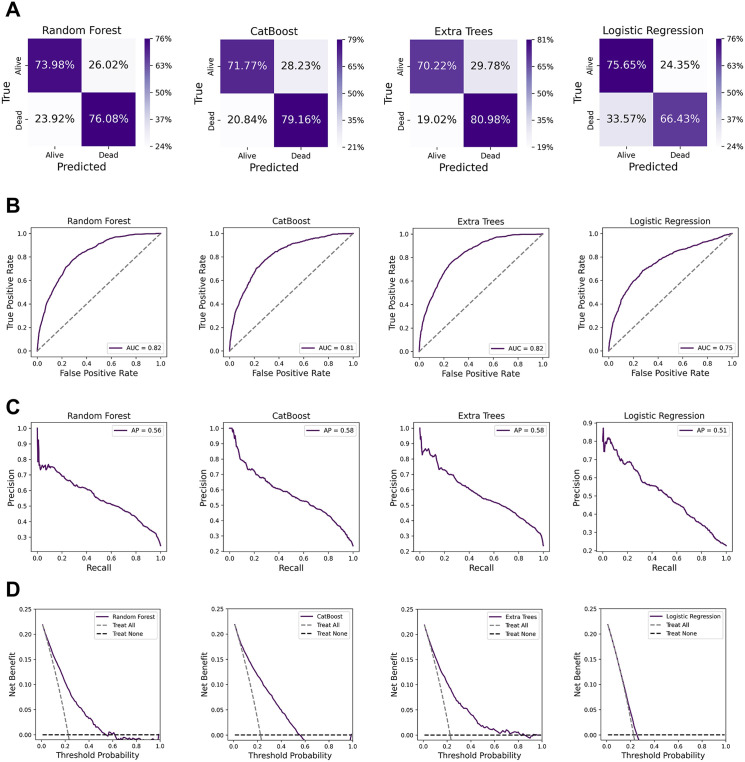
Confusion matrix (A), ROC curve (B), precision/recall curve (C) and decision curve analyses (D) for single models using the five-subdivision approach.

For the three-subdivision approach, Random Forest and Extra Trees achieved an average AUROC of 0.83, followed closely by CatBoost at 0.82 and Logistics Regression at 0.76. The corresponding AUPRC values were 0.46 for Random Forest, 0.48 for CatBoost and Extra Trees, and 0.41 for Logistic Regression. In the five-subdivision approach, Random Forest and Extra Trees both achieved an average AUROC of 0.82, while Catboost reached 0.81. The respective AUPRC values were 0.56, 0.58, 0.58 for Random Forest, CatBoost, and Extra Trees. Figs 6 and 7 present the confusion matrices, ROC curves, and precision–recall curves for all evaluated models across the three- and five-subdivision strategies. The detailed performance metrics for each subdivision are summarized in Table 2.

### Pairwise comparisons of AUC performance

Pairwise DeLong tests were performed to compare AUC performance between the stacking ensemble and each individual classifier. As shown in S3 Table, the stacking model significantly outperformed all single models, with all comparisons yielding p < 0.01. Among the individual classifiers, Random Forest and Extra Trees showed comparable performance (p = 0.12) and appeared to outperform Logistic Regression and CatBoost. S4 and S5 Tables summarize the p-value comparisons for the three-subdivision and five-subdivision strategies, respectively. In both strategies, Random Forest and Extra Trees again showed comparable performance, while both generally performed better than Logistic Regression and CatBoost. Although the stacking ensemble was not directly compared with the subdivision-based models in the pairwise statistical analyses, it nonetheless achieved the highest AUC-ROC performance overall.

### Model interpretation

The calibration curve for the stacking model (Fig 5E) suggested generally acceptable agreement between predicted probabilities and observed event frequencies. Across most of the predicted risk range, the apparent calibration curve approximated the ideal diagonal line, although some deviation was observed at the higher-risk end. The stacking model yielded a Brier score of 0.04, which was lower than those of the individual base models (0.06 for Random Forest, CatBoost, and Extra Trees; 0.17 for Logistic Regression), demonstrating markedly superior overall calibration and reliability of its probabilistic predictions (S2A Fig).

The feature importance analyses (S2B Fig) revealed distinct patterns across the four base learners. Random Forest and Extra Trees consistently ranked D-dimer, gender, reference TT, calcium and chloride among their most influential predictors, emphasizing the combined contributions of coagulation markers, demographic factors, and electrolyte balance. CatBoost assigned high importance to gender, chloride, sodium, and potassium, highlighting the dominant role of electrolyte abnormalities in outcome prediction. In contrast, Logistic Regression identified calcium, reference TT, potassium, and INR as the strongest predictors, demonstrating its preference for linear relationships involving coagulation and metabolic parameters. For the stacking ensemble (Fig 5F), which aggregates information from all four base models, Logistic Regression and CatBoost contributed the largest weights, followed by Extra Trees and Random Forest.

Across both the three-subdivision and five-subdivision approaches, consistent patterns emerged in the feature importance rankings across all four algorithms (Random Forest, CatBoost, Extra Trees, and Logistic Regression). Coagulation-related variables, including D-dimer was repeatedly identified as dominant predictors, underscoring the central role of coagulation abnormalities in risk stratification. Electrolyte and liver–kidney function markers, particularly chloride, sodium, ALT, AST, and creatinine, also ranked highly across multiple models, highlighting their contribution to metabolic and organ-dysfunction–related risk signals. Albumin, glucose, and total bilirubin appeared consistently as secondary but meaningful predictors, suggesting their supportive roles in capturing nutritional, metabolic, and hepatic status.

## Discussion

Mortality risk prediction in pediatric critical care remains a critical challenge [15]. Increased focus on ML-based risk prediction models in PICUs can be attributed to limitations of existing risk prediction tools. These generic severity scores were designed for population-level mortality assessment rather than guiding care for individual patients. Their reliance on the worst values within a fixed period can overlook the dynamic clinical course [16]. Predictive analytics using time series data has been introduced to monitor patient deterioration before clinical signs appear [17]. The incorporation of newer risk prediction models is also due to the real-time capabilities of electronic health records (EHRs). Early warning scores (EWS) have been developed to identify patients needing intervention, with various tools now in use alongside rapid response teams in hospitals. For example, the Bedside Pediatric Early Warning Score (PEWS) is widely used across the UK to detect patients at risk of acute deterioration and escalate their care [17,18]. In ICUs, patients are critically unstable and their condition fluctuates, therefore requiring swift monitoring and support [19].

In our study, disturbances in coagulation function and electrolyte balance were key predictors of mortarlity. Coagulation dysfunction was a hallmark of non-survivors, with elevated INR and D-dimer levels. Prolonged INR indicates impaired clotting factor synthesis, often due to liver failure or vitamin K deficiency, while elevated D-dimer signals uncontrolled fibrinolysis, as seen in sepsis and trauma [20,21]. Metabolic derangements, including hyperglycemia and hypertriglyceridemia were also significant predictors. Stress-induced hyperglycemia arises from cortisol and catecholamine surges, impairing immune function and endothelial integrity [22]. PICU patients routinely undergo extensive laboratory testing upon admission, and these tests provide critical insights into their physiological status. Our findings that elevated INR and D-dimer levels are associated with mortality are consistent with existing literature, further validating the prognostic value of these parameters in pediatric critical care.

In addition to coagulation abnormalities, disruptions in electrolyte balance also played a substantial role in mortality risk. Multiple studies have demonstrated that hypocalcemia, serum calcium levels below 8.5 mg/dL is linked to a higher risk of all-cause mortality at treatment initiation. For example, Yamaguchi et al., in their study reported that hidden hypocalcemia was a strong predictor of mortality, as seen in the present study [23]. Similarly, hypocalcemia has been associated with higher mortality in PICU [24]. The incidence of hypocalcemia is also quite high in PICU as evidenced by Thapar et al., in their study, which showed that approximately half of the patients admitted to the PICU had hypocalcemia [25]. Similarly, another study showed that hypocalcemia was an independent risk factor for mortality in children [26].

This study evaluated two strategies to address class imbalance in pediatric mortality prediction: stacking ensemble and data subdivision. In contrast to traditional concerns that stacking may introduce redundancy when combining multiple tree-based models, our findings demonstrated that the stacking ensemble outperformed all individual models, achieving superior AUC-ROC, AUC-PRC, calibration, and net clinical benefit. This enhanced performance suggests that the meta-classifier was able to effectively integrate complementary information from heterogeneous base learners, both linear (Logistic Regression) and nonlinear (Random Forest, CatBoost, Extra Trees)and leverage their diverse decision boundaries rather than amplifying redundancy. Consequently, the stacking framework successfully captured broader patterns within the data and demonstrated greater robustness in handling class imbalance compared with any single classifier.

In contrast, the data subdivision approach produced performance that was comparable to the single models but did not yield further improvement. Subdivision was originally designed to reduce the impact of class imbalance by generating multiple balanced datasets. However, once missingness was properly addressed through MI and oversampling was consistently applied across the full cohort, the added value of subdividing the data diminished. The single models trained on MI augmented and SMOTE balanced datasets already achieved stable discrimination and calibration, leaving limited room for subdivision to confer additional benefits. These results suggest that when missing data are appropriately handled through multiple imputation, full cohort modeling provides robust and reliable performance without the need for additional data partitioning strategies.

Compared with established PICU mortality scoring systems, recent evidence from meta analysis reports that PRISM 3 achieves an AUC of 0.84 (95 CI, 0.80 to 0.87), PIM 3 achieves an AUC of 0.82 (95 CI, 0.78 to 0.85), and PELOD 2 achieves an AUC of 0.83 (95 CI, 0.80 to 0.86) [27]. Similarly, the Phoenix criteria for pediatric sepsis and septic shock demonstrate an AUC range of 0.71 to 0.92 (95 CI, 0.70 to 0.92) [28]. In comparison with these benchmarks, our machine learning model demonstrated predictive performance that is comparable to, and in some cases exceeds, that of traditional scoring systems.

Unlike conventional tools that require multiple physiological measurements collected over defined time windows, the machine learning model developed in this study relies on routine laboratory parameters at PICU admission. This characteristic has the potential to reduce clinical burden by limiting repeated assessments while still providing accurate early risk stratification. Moreover, previous multicenter studies suggest that PRISM and PIM scores may have reduced discriminative power in regions outside North America and Europe [17]. By contrast, the approach used in our study may offer a more flexible and broadly applicable alternative for diverse healthcare settings, particularly those with limited resources or inconsistent availability of standardized monitoring protocols.

### Limitations and future directions

Despite the promising findings of this study, several limitations should be acknowledged. First, the retrospective design and reliance on a single-center dataset may limit the generalizability of the model. Additionally, the absence of vital signs and temporal clinical variables restrict the model’s ability to capture dynamic patient trajectories. Although SMOTE was applied to address class imbalance, the risk of overfitting cannot be entirely excluded. Moreover, external validation using independent datasets was not conducted, which is essential for evaluating the model’s reliability and clinical applicability. We also acknowledge that widely used prognostic scores, such as PIM-3 and PELOD-2, were not available in the public dataset employed, we conducted an indirect comparison by referencing the range of AUC values reported in other studies.

Future research should focus on validating the proposed approach in larger, multicenter cohorts to improve generalizability. Incorporating temporal features and exploring alternative resampling techniques or advanced ensemble strategies may further enhance performance. Moreover, integrating additional predictive biomarkers, such as inflammatory markers, genetic factors, or real-time physiological signal could strengthen model precision and clinical relevance. Through these refinements, machine learning-based models may evolve into practical, accurate, and scalable tools for mortality prediction and decision support in pediatric intensive care settings.

## Conclusions

Our findings indicate that stacking model significantly mitigates the inherent class imbalance in the dataset, possibly by ensuring adequate representation of minority classes (mortality cases) during model training while preserving the diversity of majority-class samples, and improves model stability as a clinical prediction model.

## Supporting information

S1 Fig Violin plots of selected clinical characteristic distribution and density. (TIF)

S2 Fig Calibration curve (A) and feature importance ranking (B) for single models. (TIF)

S3 Fig Calibration curve (A) and feature importance ranking (B) for single models using the three-subdivision approach. (TIF)

S4 Fig Calibration curve (A) and feature importance ranking (B) for for single models using the five-subdivision approach. (TIF)

S1 Table Hyperparameter search space for the machine learning models. (DOCX)

S2 Table Python packages, versions, and their corresponding functions used in data preprocessing, imputation, model development, and performance evaluation. (DOCX)

S3 Table P-values from pairwise AUC comparisons between individual models and the stacking ensemble. (DOCX)

S4 Table P-values from pairwise AUC comparisons across single models using the three-subdivision approach. (DOCX)

S5 Table P-values from pairwise AUC comparisons across single models using the five-subdivision approach. (DOCX)

## Abbreviations

AUC: Area Under the Curve
AUROC: Area Under the Receiver Operating Characteristic Curve
AUPRC: Area Under the Precision-Recall Curve
ALT: Alanine Aminotransferase
AST: Aspartate Aminotransferase
EHR: Electronic Health Record
ICU: Intensive Care Unit
PICU: Pediatric Intensive Care Unit
INR: International Normalized Ratio
Lasso: Least Absolute Shrinkage and Selection Operator
ML: Machine Learning
PELOD: Pediatric Logistic Organ Dysfunction
PIM: Pediatric Index of Mortality
PRISM: Pediatric Risk of Mortality
PT: Prothrombin Time
PTT: Partial Thromboplastin Time
ROC: Receiver Operating Characteristic
SMOTE: Synthetic Minority Oversampling Technique
TT: Thrombin Time.
