# Supplementary figures and images for: Data subdivision approach enhances machine learning-based mortality prediction in pediatric ICU patients
Source: PLoS One. 2026 Jun 16;21(6):e0349772. doi: 10.1371/journal.pone.0349772 (PMC13271752; doi:10.1371/journal.pone.0349772)

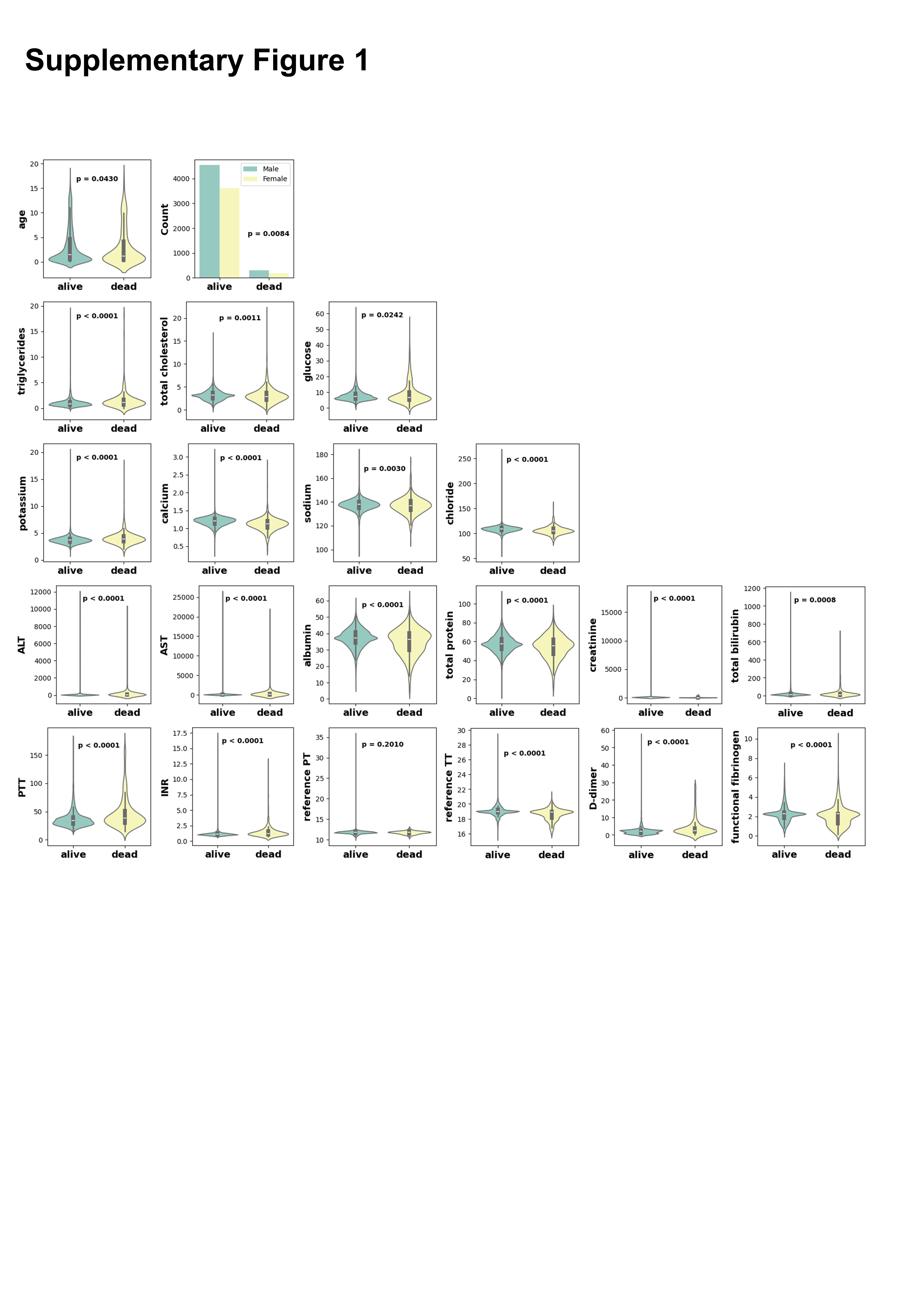

Supplement: S1 Fig — (TIF) [file pone.0349772.s001.TIF]

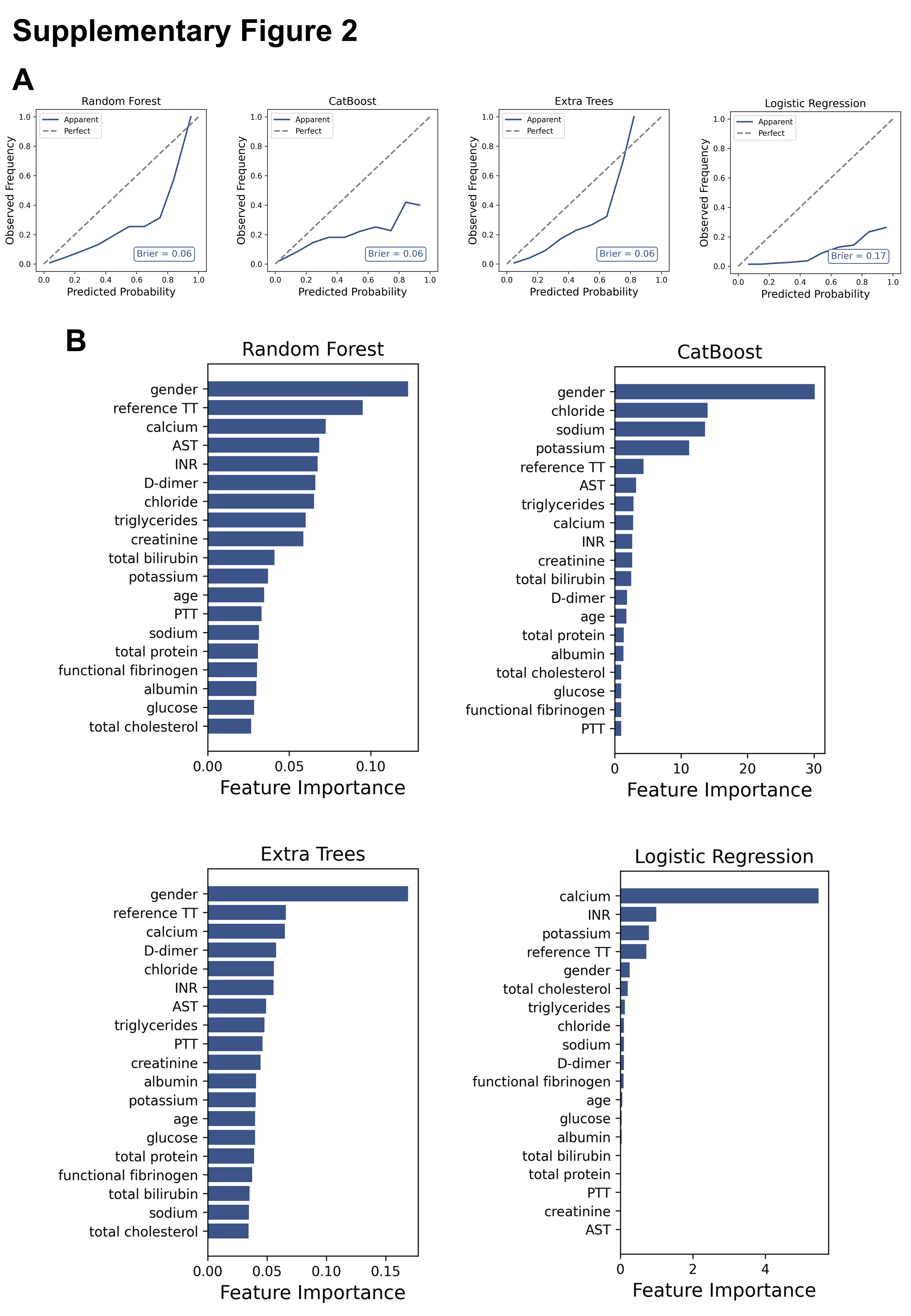

Supplement: S2 Fig — (TIF) [file pone.0349772.s002.TIF]

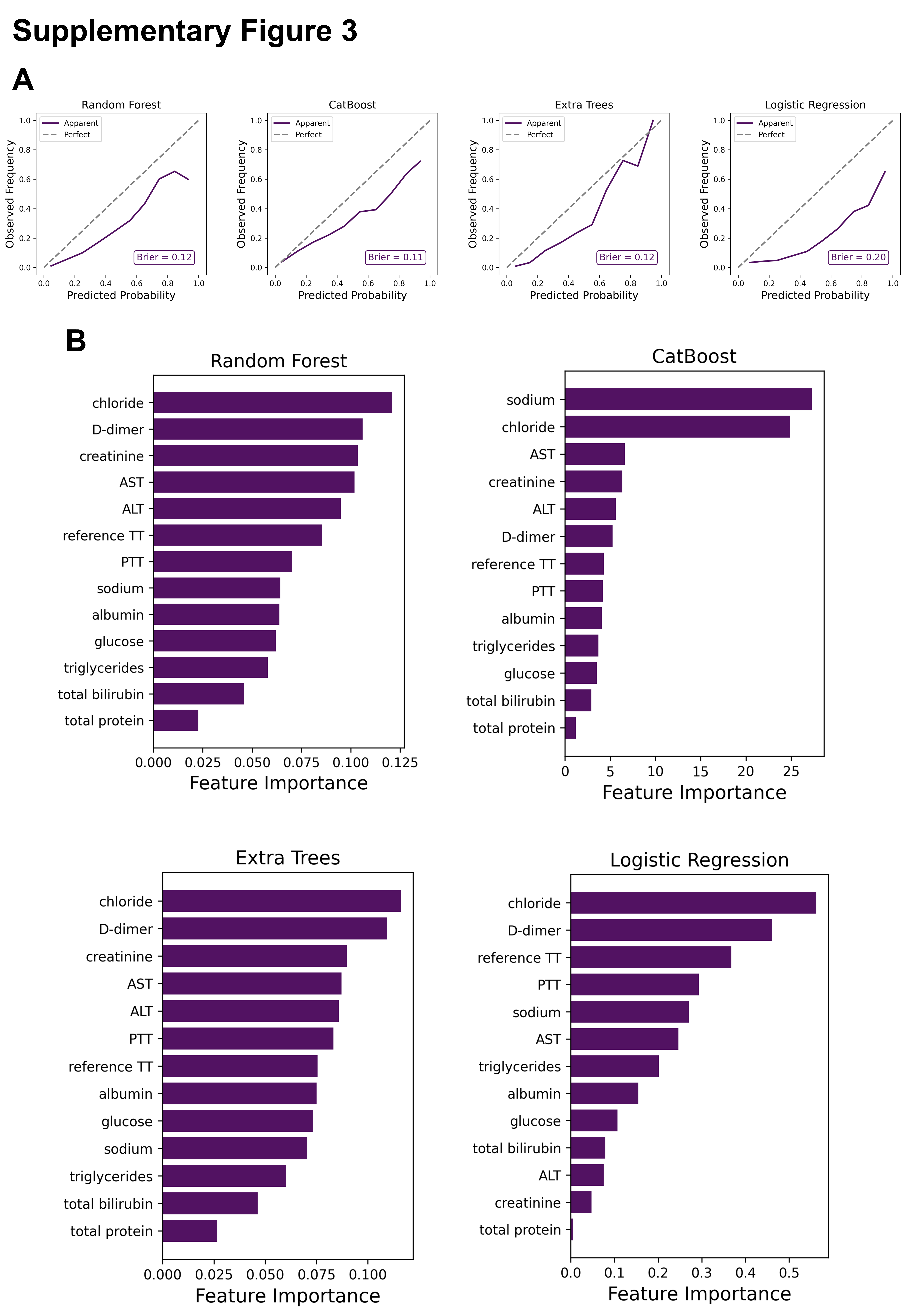

Supplement: S3 Fig — (TIF) [file pone.0349772.s003.TIF]

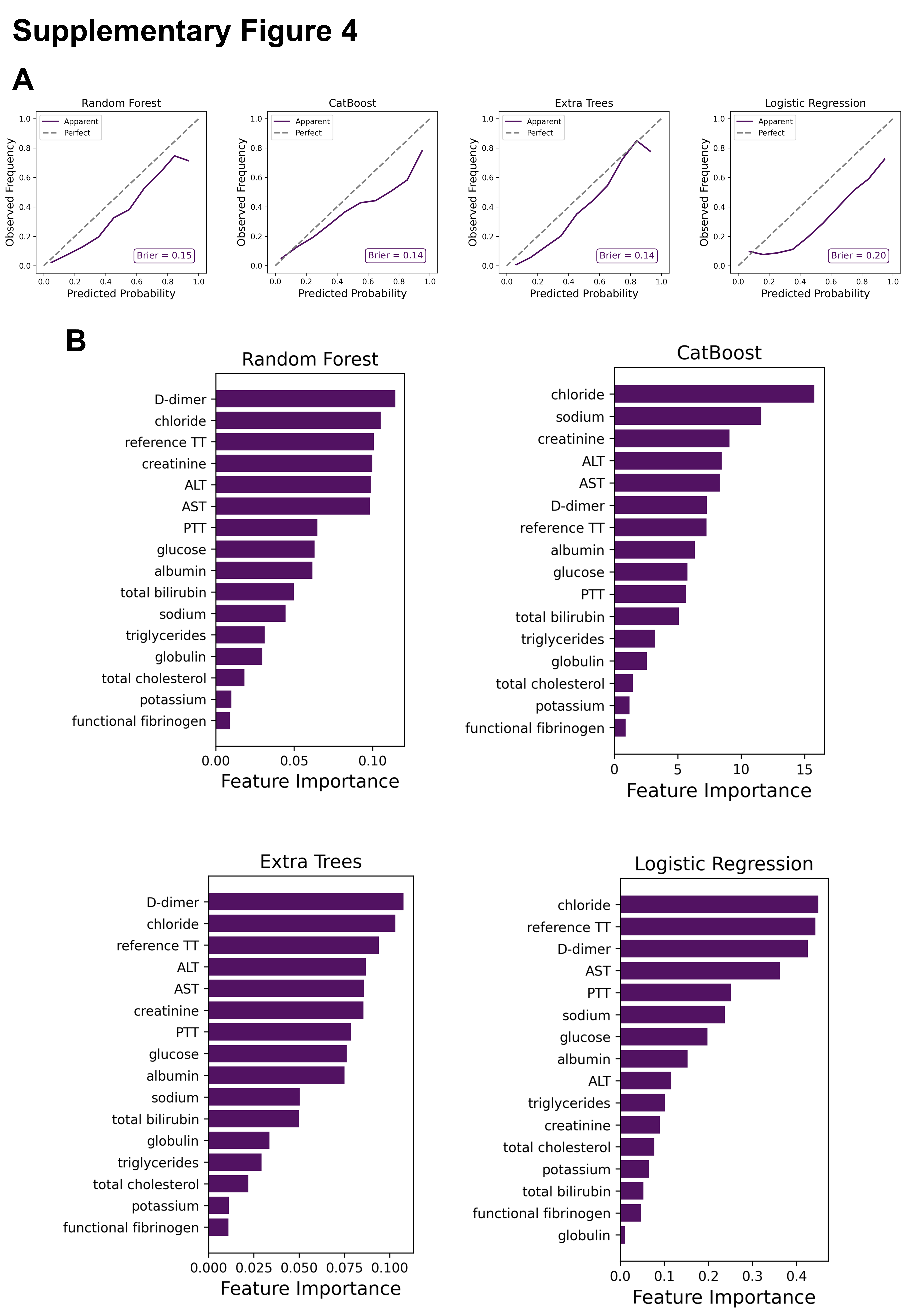

Supplement: S4 Fig — (TIF) [file pone.0349772.s004.TIF]
