# Supplementary material for: Data subdivision approach enhances machine learning-based mortality prediction in pediatric ICU patients
Source: PLoS One. 2026 Jun 16;21(6):e0349772. doi: 10.1371/journal.pone.0349772 (PMC13271752; doi:10.1371/journal.pone.0349772)
Supplement: S1 Table — (DOCX) [file pone.0349772.s005.docx]

**Supplementary Table 1.** Hyperparameter search space for the machine learning models

| **Strategy** | **Model** | **Hyperparameter** | **Candidate values** |
| --- | --- | --- | --- |
| **Single Model** | Logistic Regression | penalty | ['l1', 'l2'] |
|  |  | C | [0.01, 0.1, 1, 10] |
|  |  | max_iter | 1000 |
|  |  | solver | liblinear |
|  | Random Forest | n_estimators | [80, 120, 150] |
|  |  | max_depth | [10, 20, None] |
|  |  | max_features | ['sqrt', 'log2'] |
|  |  | min_samples_leaf | [1, 2] |
|  | CatBoost | learning_rate | [0.03, 0.05] |
|  |  | depth | [4, 5, 6] |
|  |  | iterations | [300, 400] |
|  |  | l2_leaf_reg | [3, 5, 7] |
|  |  | subsample | [0.7, 0.9, 1.0] |
|  | Extra Trees | n_estimators | [120, 150] |
|  |  | max_depth | [10, 20, None] |
|  |  | max_features | ['sqrt'] |
|  |  | min_samples_leaf | [1, 2] |
| **Stacking model** | Gradient Boosting | n_estimators | [100, 150, 200] |
|  |  | learning_rate | [0.03, 0.05, 0.1] |
|  |  | max_depth | [2, 3, 4] |
|  |  | subsample | [0.7, 0.8, 1.0] |
|  |  | min_samples_split | [2, 4] |
| **Three-subdivision** | Logistic Regression | penalty | ['l1', 'l2'] |
|  |  | C | [0.01, 0.1, 1, 10] |
|  |  | max_iter | 1000 |
|  |  | solver | liblinear |
|  | Random Forest | n_estimators | [80, 120, 150] |
|  |  | max_depth | [10, 20, None] |
|  |  | max_features | ['sqrt', 'log2'] |
|  |  | min_samples_leaf | [1, 2] |
|  | CatBoost | learning_rate | [0.03, 0.05] |
|  |  | depth | [4, 5, 6] |
|  |  | iterations | [300, 400] |
|  |  | l2_leaf_reg | [3, 5, 7] |
|  |  | subsample | [0.7, 0.9, 1.0] |
|  | Extra Trees | n_estimators | [120, 150] |
|  |  | max_depth | [10, 20, None] |
|  |  | max_features | ['sqrt'] |
|  |  | min_samples_leaf | [1, 2] |
| **Five-subdivision** | Logistic Regression | penalty | ['l1', 'l2'] |
|  |  | C | [0.01, 0.1, 1, 10] |
|  |  | max_iter | 1000 |
|  |  | solver | liblinear |
|  | Random Forest | n_estimators | [80, 120, 150] |
|  |  | max_depth | [10, 20, None] |
|  |  | max_features | ['sqrt', 'log2'] |
|  |  | min_samples_leaf | [1, 2] |
|  | CatBoost | learning_rate | [0.03, 0.05] |
|  |  | depth | [4, 5, 6] |
|  |  | iterations | [300, 400] |
|  |  | l2_leaf_reg | [3, 5, 7] |
|  |  | subsample | [0.7, 0.9, 1.0] |
|  | Extra Trees | n_estimators | [120, 150] |
|  |  | max_depth | [10, 20, None] |
|  |  | max_features | ['sqrt'] |
|  |  | min_samples_leaf | [1, 2] |
