# Supplementary material for: Data subdivision approach enhances machine learning-based mortality prediction in pediatric ICU patients
Source: PLoS One. 2026 Jun 16;21(6):e0349772. doi: 10.1371/journal.pone.0349772 (PMC13271752; doi:10.1371/journal.pone.0349772)
Supplement: S2 Table — (DOCX) [file pone.0349772.s006.docx]

**Supplementary Table 2** Python packages, versions, and their corresponding functions used in data preprocessing, imputation, model development, and performance evaluation.

| Category | Package | Version | Purpose |
| --- | --- | --- | --- |
| Core Numerical Libraries | numpy | 1.24.3 | Numerical computations and arrays |
|  | pandas | 2.0.3 | Data manipulation and preprocessing |
| Machine Learning Models | scikit-learn | 1.3.0 | Classical ML models, metrics, and utilities |
|  | catboost | 1.2 | CatBoost classifier |
|  | xgboost | 1.7.6 | XGBoost model (if used) |
|  | GradientBoostingClassifier (sklearn) | 1.3.0 | Stacking/ensemble components |
| Imputation & Balancing | IterativeImputer (sklearn) | 1.3.0 | Multiple Imputation (MICE) |
|  | imbalanced-learn | 0.11.0 | SMOTE oversampling |
| Visualization | matplotlib | 3.7.1 | Plotting ROC, PR, calibration curves |
| Utilities / Output | openpyxl | 3.1.2 | Excel output |
