# Supplementary material for: Data subdivision approach enhances machine learning-based mortality prediction in pediatric ICU patients
Source: PLoS One. 2026 Jun 16;21(6):e0349772. doi: 10.1371/journal.pone.0349772 (PMC13271752; doi:10.1371/journal.pone.0349772)
Supplement: S4 Table — (DOCX) [file pone.0349772.s008.docx]

**Supplementary Table 4** P-values from pairwise AUC comparisons across three-subdivision approach.

| Strategy | | Three-subdivision | | | |
| --- | --- | --- | --- | --- | --- |
|  |  | Logistic Regression | Random Forest | CatBoost | Extra Trees |
| Three-subdivision | Logistic Regression |  |  |  |  |
|  | Random Forest | < 0.01 |  |  |  |
|  | CatBoost | < 0.01 | < 0.01 |  |  |
|  | Extra Trees | < 0.01 | 0.18 | 0.01 |  |
